# Bowel Perforation after Extracorporeal Wave Lithotripsy: A Review of the Literature

**DOI:** 10.3390/jcm12031052

**Published:** 2023-01-29

**Authors:** Sofia Fontanet, Alba Farré, Oriol Angerri, Andrés Kanashiro, Edgar Suquilanda, Jesús Bollo, Maria Gallego, Francisco Maria Sánchez-Martín, Félix Millán, Joan Palou, Diana Bonnin, Esteban Emiliani

**Affiliations:** 1 Department of Urology, Fundación Puigvert, Autonomous University of Barcelona, 08025 Barcelona, Spain; 2 Department of General Surgery, Sant Pau Hospital, Autonomous University of Barcelona, 08025 Barcelona, Spain; 3 Department of Radiology, Fundación Puigvert, Autonomous University of Barcelona, 08025 Barcelona, Spain

**Keywords:** ESWL, lithotripsy, stone, urology, bowel perforation

## Abstract

Introduction: Extracorporeal wave lithotripsy (ESWL) is considered a first-line treatment for renal and ureteral stones up to 10–20 mm in diameter. Complications are uncommon, with a reported rate of 0–6% in the literature. Bowel perforation has only been described in a few case reports but requires rapid diagnosis and treatment. Methods: A review of the literature from PubMed/Medline, Embase, Cochrane, and Web of Science databases was performed including studies reporting bowel perforation secondary to ESWL between January 1990 and June 2022. Results: We found 16 case reports of intestinal perforation in the literature. Although some patients had previously undergone abdominal surgery or had inflammatory intestinal disease, others were without comorbidities that could lead to complications. Abdominal pain was the main symptom and imaging was required to confirm the diagnosis, which usually necessitated a surgical intervention. As regards the ESWL technique, it appears that the combination of a high energy level and the prone position constitutes a risk factor for these rare complications. At the authors’ centre, only one case has been reported among 24,000 ESWL procedures over 20 years: A 59-year-old female who underwent ESWL for a distal right ureteral stone presented acute abdominal pain and free intraperitoneal pelvic fluid on ultrasound. A CT scan revealed a small bowel perforation requiring open laparotomy with primary closure. Conclusions: In conclusion, although bowel perforation after ESWL is rare, progressive abdominal pain with tenderness at physical examination requires proper imaging evaluation to exclude bowel perforation and prompt intervention if required.

## 1. Introduction

Since its introduction in the 1980s [1], extracorporeal wave lithotripsy (ESWL) has been shown to be a safe, effective, and non-invasive treatment for renal and ureteral stones up to 10–20 mm in diameter [2]. In the last two decades, ESWL technology has evolved, especially as regards the quality of shock wave generators, coupling, and stone location, making it possible to optimize results while reducing failure rates [1,3].

Complications are infrequent, occurring in 0–6% of patients according to the literature [4]. The vast majority appear in the short term and are Clavien-Dindo grades I and II complications requiring pharmacological treatment. Among these, the most common are renal colic (28%), ureteral obstruction (4%), sepsis (1%), renal function impairment (0.4%), and renal hematoma (0.4%) [5,6,7]. Other severe complications, commonly classified as Clavien-Dindo grades III and IV and usually requiring surgical management, include hepatic hematoma, pancreatitis, cardiac arrhythmia, aortic aneurysm rupture, spleen rupture, and bowel perforation [7,8,9,10,11]. While gastrointestinal damage has been described as a rare complication of ESWL, the actual incidence is unknown. Bowel perforations are uncommon, having been reported in only 16 cases in the literature to our knowledge [12,13,14,15,16,17,18,19,20,21,22,23,24,25,26,27]. Here, we present a review of the literature, with the addition of the only case we have had in our hospital in 20 years.

## 2. Methods

A review of the literature was performed from PubMed, Medline, Embase, Cochrane and Web of Science databases including studies published between January 1990 and June 2022. Two reviewers (A.F. and S.F) conducted the research in the databases previously mentioned. The search was performed by using the combination of the words “ESWL” and “bowel” or “intestinal” and “perforation” or “complications” and “urinary” or “urologic” and “stones”. Those keywords led to 16 results in which ESWL for urologic stones were complicated by colonic or small bowel perforation.

A data analysis was performed to evaluate risk factors and preventive measures.

## 3. Results

A total of 16 cases have been reported in the literature since 1997 [12,13,14,15,16,17,18,20,21,22,23,24,25,26,27]. Demographics are summarised in Table 1.

Twelve patients were male, three were female, and one was of unknown gender. Patient age ranged from 29 to 78 years.

The interval between the ESWL procedure and the onset of symptoms varied between a few hours and 2 days. All patients presented at the emergency department with a main complaint of acute abdominal pain with tenderness at physical examination. Moreover, fever was present in one-third (5/15) of the cases.

During the 1990s, a KUB X-ray was performed for diagnostic imaging, whereas since then CT scan has been the main diagnostic tool. 

A surgical approach with bowel repair was needed in 15 cases due to the high morbidity of intestinal perforation or hemodynamic instability. The remaining case was managed conservatively without further complications; this treatment strategy was chosen because the patient was hemodynamically stable without signs of peritonitis.

The location of the perforation varied according to the ESWL treatment area. In six of the sixteen cases [14,15,17,18,20,22], the perforation occurred in the colon, while in the remaining nine cases the small bowel was affected.

Perilesional findings during surgical exploration [13,20,23] included necrotic areas, bruised areas, congestion, and inflammation.

Regarding the stone location prior to ESWL, there was only one renal stone [20], with ureteral stones being the most common. Eight studies reported stone size, and in only one case was the stone larger than 10 mm [27].

The prone position was used in 11 cases, a combination of prone and supine in one, and supine in two [14,16]; in two studies the position was not reported. The total number of shocks delivered varied from 2500 to 6000. Table 2 summarizes the ESWL characteristics.

At the authors’ institution, just one case of bowel perforation has been reported after 24,000 ESWL procedures performed over 20 years; this patient needed open surgery and primary closure of the wound, as further discussed below.

## 4. Discussion

The incidence of bowel perforation after ESWL is unknown. Our literature review revealed reports of only 16 cases, although cases of gastrointestinal damage other than perforations have been described in single case series. These other complications have included gastric or duodenal erosions, ureterocolic fistula, gastrojejunal anastomotic dehiscence, small bowel obstruction and strangulation, cecal ulcers, sigmoid colon hematoma, peripancreatic hematoma, peripancreatic abscess, pancreatitis, and rectal bleeding [14,28,29].

Interestingly, most cases of intestinal perforation after ESWL are seen in males, and patient age varies greatly. The predominance of male cases can perhaps be ascribed to the higher incidence of urolithiasis in the male population. Although some of the patients that we identified had a medical history of abdominal surgeries or intestinal inflammatory disease, most had no relevant past medical history or risk factors. Nonetheless, patient selection should be made carefully, or complications are likely to occur in these cases.

In this review, in the vast majority of cases (13/16) only one ESWL session was performed before intestinal perforation occurred. However, in one case it was reported that the stone was initially in the renal pelvis, where the first ESWL session was conducted [12]. Additionally, in two other cases [19,20] more previous ESWL sessions were described but it was not clearly specified if they were performed for the same stones. The interval from the procedure to the onset of symptoms varied between a few hours and 2 days, with the typical presentation being acute abdominal pain with tenderness at physical examination. Patient’s management was surgical from the beginning in 12 cases because of clinical signs of peritonitis [12,13,14,17,18,20,21,24,27]. Only in four cases [19,22,23,25] was conservative management with antibiotic initially preferred. In three of them, due to clinical worsening, patients had to undergo surgery between 24 and 48 h. In the other case [19], the patient remained hemodynamically stable and a control CT scan was performed at 7 days with signs of improvement.

Finally, there was one case [16] where obstructive stones was first suspected, so a double J stent was placed. Then, due to clinical signs of peritonitis, a CT was performed revealing signs of bowel perforation, so the patient finally underwent surgery.

Therefore, a surgical approach with bowel repair was required in all cases except one patient [19]. There were no fatal outcomes and clinical recovery after surgery was complete.

ESWL treatment can affect either the small bowel or the colon, depending on the stone location. In our review, the mean stone diameter was 8.2 mm, and in only one case did the stone diameter exceed 10 mm. As regards the ESWL technique, it appears that the combination of a high energy level and the prone position constitutes a risk factor for these rare complications.

The single case of bowel perforation after ESWL at the authors’ institution, where 24,000 ESWL procedures have been performed over the past 20 years, was in a 59-year-old female with a past medical history of dyslipidemia, chronic bronchitis, and a C-section. Initially, in the context of colic lumbar pain, she had been diagnosed as having a 17 mm right distal ureteral stone at her referring hospital. As the patient presented with moderate hydronephrosis, a right nephrostomy tube was placed, and she was referred to our centre for definitive treatment of the stone. Three weeks later, under sedation, she underwent a first ESWL session, resulting in partial fragmentation. The patient was placed in the supine position on a Lithoskop lithotripter (Siemens, Munich, Germany), which delivered 4500 shocks to the stone with a total energy of 175 joules.

Four hours after the procedure, the patient returned to the emergency department due to discomfort and intense right abdominal pain. Physical examination revealed painful palpation and localized tenderness at the flank and right iliac fossa. A blood test showed 15,000 × 10^9^/L leukocytosis with neutrophilia (82%). She did not present fever, but abdominal pain was worsening despite analgesia. An abdominal ultrasound revealed a small amount of free intraperitoneal fluid. A CT scan with contrast was performed, resulting in the diagnosis of a small bowel perforation at the level of the terminal ileum with free abdominal fluid and a small amount of pneumoperitoneum.

The general surgery department evaluated the case and suggested exploratory laparoscopy, which revealed diffuse purulent peritonitis with possible perforation in the mesenteric margin of the terminal ileum as well as a hematoma in the mesojejunum and two lesions in terminal ileum serosa close to the main lesion. As the lesions could not be clearly assessed, the decision was taken to perform a mini-laparotomy, which confirmed the perforation identified by laparoscopy. Simple closure was performed without intestinal resection.

During the postoperative follow-up, the patient remained hemodynamically stable and afebrile, progressively tolerating an oral diet. She was discharged from the hospital 5 days after the intervention. Follow-up at the first month was unremarkable. Two months later, a right ureteroscopy with laser dusting of the stone and double J stent placement was performed without abnormal intraoperative findings. During endoscopy, no alterations of the ureteral wall were found. Three weeks later, the double J stent was removed, and the patient has since been stone free with no complications.

Risk factors for bowel perforation due to ESWL reported in the literature are previous abdominal surgery, prone position, placement of the ureter too close to the intestine, and the use of high energy levels [14].

Prompt diagnosis of a bowel perforation is crucial to avoid further complications. Initial physical examination to assess abdominal acute pain and tenderness should be complemented with a general blood test and the performance of a CT scan. Once the diagnosis has been established, surgery seems to be an effective treatment, with good patient recovery. Stone treatment can be offered at the same time with ureterotomy and stone removal, but in most cases, treatment is deferred.

## 5. Conclusions

Although bowel perforation is rare, and renal colic is the main complication after ESWL, patients with acute abdominal pain few hours after the procedure should undergo a proper physical exploration to search for peritoneal irritation. Furthermore, it usually requires accurate imaging evaluation. Imaging may enable the physician to rule out an intestinal complication or to initiate prompt intervention if required, bearing in mind the high morbidity of the condition. The combination of a high energy level and the prone position seems to constitute a risk factor for this rare complication.

## Figures and Tables

**Table 1:** Demographic data.

Publication	Year	Intestine Location Affection	Gender	Age (y)	Past Medical History	Symptoms	Stone Size	Lithiasis Location	Diagnostic Imaging
Geh et al. [12]	1997	Terminal ileum	M	55	NA	Right-sided abdominal pain	NA	Right ureter	Chest radiography
Holmberg et al. [13]	1997	Mid portion of the small bowel (ileum)	M	68	Asthma	Abdominal pain and signs of localized peritonitis	6 × 4 mm	Right mid ureter	Plain abdominal X-ray
Castillon et al. [20]	1999	Left transverse colon	M	29	Bilateral kidney stones treated by ESWL (23,500 SW)	Acute abdominal pain	9 × 5 mm	Left renal pelvis	Abdominal X-ray
Kurtz et al. [21]	1999	Small bowel	M	57	Prostate cancer and bilateral scrotal orchiectomy	Right-sided abdominal pain and incipient signs of peritonitis	NA	Right reno-ureteral	Abdominal and chest X-ray and US
Lipay et al. [22]	2000	Distal sigmoid colon	M	32	NA	Pain in the left iliac fossa and diarrhea	8 mm	Left distal ureter	Abdominal X-ray
Olsson et al. [23]	2000	Ileum	M	44	NA	Left abdominal pain and mild ecchymosis	6 × 3 mm	Left mid ureter	CT
Klug et al. [24]	2001	Small bowel	M	60	Billroth II penis amputation	Intense abdominal pain	NA	Right distal ureter (L3–L4)	US
Kajikawa et al. [25]	2001	Jejunum	M	69	Graft replacement for bilateral iliac aneurysm	Left lower abdominal pain	NA	Left ureter	CT
El-Faqih [26]	2002	Proximal ileum	M	38	NA	Central abdominal pain and fever	10 × 6 mm	Right mid ureter	Abdominal and chest X-ray
Rodriguez Netto et al. [27]	2003	Ileum	F	51	History of stone disease	Severe abdominal pain and nausea, vomiting and fever	14 × 8 mm	Left mid ureter	Contrast CT
Maker et al. [14]	2004	Cecum	M	44	Congenital hypospadias, ureteral strictures, recurrent nephrolithiasis	Abdominal and flank pain and nausea, vomiting	NA	Right proximal ureter	Abdominal X-ray and CT
Cardinaux et al. [15]	2009	Cecum	F	78	Anterior pelvic exenteration with ureterosigmoidostomy	Acute abdominal tenderness and fever	5 mm	Right distal ureter	CT
Chhor et al. [16]	2009	Jejunum	M	33	Crohn’s disease complicated by jejunal abscess	Left flank abdominal pain and fever	NA	Right ureter	CT
Kurz et al. [17]	2009	Descending colon	M	40	NA	Left quadrant abdominal pain	4 × 8 mm	Left ureter L4	US
Arrebal et al. [18]	2010	Ascending colon	-	34	NA	Abdominal pain + fever	NA	Right ureter	NA
Galeano et al. [19]	2022	Small bowel	F	56	History of recurrent reno-ureteral colic requiring several sessions of ESWL	Left hemi-abdominal pain and nausea, vomiting and haematuria	NA	NA	CT

y = years; NA = not available; M = male; F = female; ESWL = External Shock Wave Lithotripsy; US = ultrasound; CT = computed tomography; SW = shock waves.

**Table 2:** Characteristics of ESWL.

Publication	Year	Position	Energy	Number of Shock Waves	Lithotripter
Geh et al. [12]	1997	Prone	NA	NA	NA
Holmberg et al. [13]	1997	Prone	6.5 kV	4500	Siemens Lithostar Plus
Castillon et al. [20]	1999	Prone	8–9 kV	4500	Storz Modulith
Kurtz et al. [21]	1999	Prone and supine	6 kV	4000	Siemens Lithostar Plus
Lipay et al. [22]	2000	Prone	7 kV	3000	NA
Olsson et al. [23]	2000	Prone	6 kV	4000	Siemens Lithostar with shock tube C
Klug et al. [24]	2001	NA	NA	5000	Modulith SL 10
Kajikawa et al. [25]	2001	Prone	NA	NA	NA
El-Faqih [26]	2002	Prone	6.5 kV	4000	Siemens Lithostar Plus
Rodriguez Netto et al. [27]	2003	Prone	4–6 kV	6000	Siemens Lithostar SWS-C
Maker et al. [14]	2004	Supine	6.5 kV	2500	NA
Cardinaux et al. [15]	2009	Prone	6–7 kV	3000	Doli S lithotripter
Chhor et al. [16]	2009	Supine	NA	3000	Dornier Delta II
Kurz et al. [17]	2009	Prone	6 kV	4000	Siemens lithostar modularis
Arrabal et al. [18]	2010	Prone	200 J	3400	Dornier Doly EMSE 220F-XXP
Galeano et al. [19]	2022	NA	NA	NA	NA

NA = not available; kV = kilovolts; J = joules; SL = Siemens Lithostar; SWS-C = Shock Wave System C.

## Data Availability

Data was extracted directly by the cited publications and by analysing our case report.
